# The salvage role of allogeneic hematopoietic stem-cell transplantation in relapsed/refractory diffuse large B cell lymphoma

**DOI:** 10.1038/s41598-023-44241-0

**Published:** 2023-10-15

**Authors:** Gi-June Min, Young-Woo Jeon, Tong Yoon Kim, Daehun Kwag, Byung-Su Kim, Joonyeop Lee, Jong Hyuk Lee, Sung-Soo Park, Silvia Park, Jae-Ho Yoon, Sung-Eun Lee, Byung-Sik Cho, Ki-Seong Eom, Yoo-Jin Kim, Seok Lee, Hee-Je Kim, Chang-Ki Min, Jong Wook Lee, Seok-Goo Cho

**Affiliations:** 1 Department of Hematology, Seoul St. Mary’s Hematology Hospital, College of Medicine, The Catholic University of Korea, Banpo-daero 222, Seocho-gu, Seoul, Republic of Korea; 2 Department of Hematology, Yeouido St. Mary’s Hematology Hospital, College of Medicine, The Catholic University of Korea, 10, 63-ro, Yeongdeungpo-gu, Seoul, Republic of Korea; 3 Department of Hematology, Eunpyeong St. Mary’s Hematology Hospital, College of Medicine, The Catholic University of Korea, 1021, Tongil Ro, Eunpyeong-gu, Seoul, Republic of Korea

**Keywords:** Haematological cancer, Haematopoietic stem cells

## Abstract

To clarify the role of allogeneic hematopoietic stem-cell transplantation (allo-HSCT) in the chimeric antigen receptor T-cell therapy era, we analyzed the clinical characteristics and outcomes of 52 patients treated with allo-HSCT with relapsed/refractory diffuse large B cell lymphoma. Most enrolled patients had previously undergone intensive treatments, the median number of chemotherapy lines was 4, and the median time from diagnosis to allo-HSCT was 27.1 months. Patients were divided into remission-achieved (n = 30) and active-disease (n = 22) groups before allo-HSCT. Over a median follow-up period of 38.3 months, overall survival (OS) and event-free survival (EFS) rates were 38.4% and 30.6%, respectively. The cumulative incidence of relapse (CIR) and the non-relapsed mortality (NRM) were 36.7% and 32.7%, respectively. OS, EFS, and graft-versus-host disease-free, relapse-free survival (GRFS) outcomes were significantly superior in the remission-achieved group with lower CIR. In a multivariate analysis, a shorter interval from diagnosis to allo-HSCT reflected relatively rapid disease progression and showed significantly poor OS and EFS with higher CIR. Patients with active disease had significantly lower EFS, GRFS, and higher CIR. Previous autologous stem-cell transplantation was associated with better GRFS. Allo-HSCT is an established modality with a prominent group of cured patients and still has a role in the CAR T-cell era, particularly given its acceptable clinical outcomes in young patients with chemo-susceptible disease.

## Introduction

Survival outcomes of non-Hodgkin lymphoma (NHL), from indolent to aggressive subtypes, improve dramatically after introduction of rituximab-based chemoimmunotherapy^1,2^. Diffuse large B-cell lymphoma (DLBCL) is the most common subtype of NHL in Korea and is relatively curable. However, 30% of patients fail to achieve complete remission (CR) and are eventually diagnosed with relapsed/refractory (R/R) DLBCL^3^. The standard of care for patients with R/R DLBCL has traditionally been intensive high-dose chemotherapy, followed by autologous hematopoietic stem-cell transplantation (auto-HSCT); however, the SCHOLAR-1 study presented disappointing outcomes in the objective response rate (ORR, 26%) and median overall survival (OS, 6.3 months), highlighting the need for more effective R/R DLBCL therapies^3^. Therefore, allogeneic HSCT (allo-HSCT) was conventionally considered a treatment option with curative potential for patients who failed to achieve CR after multi-line chemotherapies and/or auto-HSCT; this was prior to the emergence of chimeric antigen receptor (CAR) T-cell therapy and other novel agents^4–6^. Conditioning regimens, alternative donor selections, and related allo-HSCT procedures constantly evolved over decades, and OS reached approximately 60%^7–9^. These improved survival outcomes following allo-HSCT were thought to be based on using tumor-free grafts and benefited from a graft-versus-lymphoma (GVL) effect. However, adverse events associated with allo-HSCT, such as acute or chronic graft-versus-host disease (GVHD) and various infectious complications, decrease quality of life and increase non-relapsed mortality (NRM). Moreover, CAR T-cell therapy for patients with R/R DLBCL after a second-line treatment failure showed favorable long-term survival and the potential to cure the disease^10–13^. Therefore, to clarify the optimal role of allo-HSCT in the CAR T-cell therapy era, we analyzed the clinical characteristics and outcomes in a long-term follow-up study of allo-HSCT-treated patients with R/R DLBCL.

## Materials and methods

### Patient enrollment and treatment strategy

In this retrospective study, we enrolled 52 patients with R/R DLBCL who underwent allo-HSCT between August 2009 and December 2020 at the Catholic Hematology Hospital. The histopathological diagnosis was reviewed based on morphological studies with immunohistochemical analysis performed by an experienced pathologist, and staging was based on the Ann Arbor system. All enrolled patients had initially undergone 6–8 cycles of R-CHOP chemotherapy, which consisted of intravascular rituximab 375 mg/m^2^, cyclophosphamide 750 mg/m^2^, doxorubicin 50 mg/m^2^, and vincristine 1.4 mg/m^2^ on day 1 and oral prednisolone 100 mg/m^2^ from days 1–5, as first-line treatment with or without frontline autologous HSCT. However, if disease progression was identified during treatment, either relapsed, primary refractory, or failed to reach at least partial remission (PR) after the end of the first-line treatment, we changed to salvage chemotherapy regimens such as DHAP (dexamethasone, high-dose cytarabine, and cisplatin), DL-ICE (dexamethasone, l-asparaginase, ifosfamide, carboplatin, and etoposide), or ESHAP (etoposide, methylprednisolone, high-dose cytarabine, and cisplatin). The detailed treatment protocol for patients with DLBCL in our institution has been described previously^14–16^. All retrospective analyses were performed after receiving approval from the Institutional Review Board (IRB) of Seoul St. Mary’s Hospital (XC23RADI0045) and following IRB guidelines and the tenets of the Declaration of Helsinki. Because the study was a retrospective analysis of anonymized routinely-collected data the IRB of Seoul St. Mary’s Hospital waived the requirement for informed consent.

### Transplant procedures

Patients who decided to undergo allo-HSCT received either myeloablative conditioning (MAC) or reduced-intensity conditioning (RIC) regimens determined by the attending physician. The MAC regimen comprised etoposide 15 mg/kg/day for 2 days, cyclophosphamide 60 mg/kg/day for 2 days, and total body irradiation (TBI) 300 cGy for 4 days. The RIC regimen consisted of fludarabine 30 mg/m^2^/day for 6 days, melphalan 70 mg/kg/day for 1 day, and 800 cGy of fractionated TBI, and is a unique regimen used in our institution for reinforcing lymphoablative activities^17,18^. Two consecutive days of anti-thymocyte globulin (ATG) 1.25 mg/kg/day administration were added for GVHD prophylaxis in cases of human leukocyte antigen (HLA) well-matched, unrelated, or mismatched donor. A calcineurin inhibitor (tacrolimus for unrelated or haploidentical donor and cyclosporin for the matched related donor) plus a short course of methotrexate on days 1, 3, 6, and 11 post-allo-HSCT were also administered for GVHD prophylaxis. In addition, acyclovir and itraconazole were administered during the transplantation process, and after engraftment for viral and fungal infection prevention, and we maintained a 6-month course of oral trimethoprim-sulfamethoxazole for *Pneumocystis jirovecii* pneumonia prophylaxis and a 1-year course of acyclovir for herpes. Calcineurin inhibitors were rapidly tapered from 3 months post-allo-HSCT and discontinued after approximately 6 months.

### Response evaluation

We defined CR as a complete palpable mass regression to the normal size on computed tomography (CT) scan with negative uptakes on 18-fluorodeoxyglucose (FDG) positron emission tomography (PET). PR was defined as achievement of at least a 50% reduction of masses observed on CT scan and positive FDG-PET findings without newly developed lesions. At the time of allo-HSCT, patients were divided into CR, PR, and active-disease groups based on disease status. The active-disease group included patients with primary refractory, chemo-resistant disease, and patients who relapsed after first-line therapy, and patients with stable disease (SD) or progressive disease (PD) after either first-line or salvage therapy. PD was defined as either an increase in the lesion size by > 25% compared with the pretreatment lesion size or the appearance of new lesions. SD was defined as a disease status that has not reached either PD or PR/CR, and relapse was defined as the PD in PR or the appearance of new lesions in patients with CR. All patients underwent response evaluation 3 months after allo-HSCT and at six-month intervals thereafter for 5 years, which included neck, chest, abdomen/pelvic CT, FDG-PET CT, and bone marrow (BM) biopsy with donor stem-cell engraftment checked using short tandem repeat analysis. Acute and chronic GVHD were diagnosed and graded according to the system of Glucksberg/Tomas and the 2014 National Institutes of Health consensus guidelines^19,20^. GVHD-free, relapse-free survival (GRFS) was defined as survival from the day of allo-HSCT to acute GVHD with grade III–IV, overall moderate-to-severe chronic GVHD, relapse, progression, or death from any cause^21^.

### Statistical analysis

Categorical and continuous variables were analyzed using the chi-square test (or Fisher’s exact test as a non-parametric method) and Student’s *t*-test (or Wilcoxon rank-sum test as a non-parametric method), respectively. Curves of OS, event-free survival (EFS), and GRFS were plotted using the log-rank test, and the cumulative incidence was used to estimate the probability of cumulative incidence of relapse (CIR) and NRM. The CIR and NRM events competed. All survival outcome measures were calculated from the time of allo-HSCT. Univariate analysis variables were selected based on previous literature on known or potentially affecting survival outcomes, and factors presenting statistical significance (*p* < 0.05) in the univariate analysis were subjected to multivariate analysis using a Cox regression model for OS, EFS, and GRFS. Fine–Gray proportional hazard regression model for competing events was used for multivariate analysis of CIR and NRM. All statistical analyses were conducted using R-software (version 4.3.1., R Foundation for Statistical Computing, Vienna, Austria, 2023), and statistical significance was set at *p* < 0.05 (two-tailed).

## Results

### Baseline characteristics

A total of 52 adult patients with R/R DLBCL were identified and enrolled. The median age at diagnosis was 45 years (range, 17–63), with marginal male predominance (n = 30, 57.7%). 36 patients (69.2%) were identified as having an advanced-stage disease (Ann Arbor stage III–IV), whereas 15 (28.8%) suffered from B symptoms. Thirteen patients (25.0%) had BM involvement of DLBCL at the time of diagnosis, and 8 of 13 (61.5%) had complex karyotypes. All enrolled patients were classified as low (n = 15, 28.8%), low-intermediate (n = 13, 25.0%), high-intermediate (n = 14, 26.9%), and high (n = 10, 19.2%) risks based on international prognostic index (IPI) risk classification. Four patients had a double-hit mutation (7.7%), and according to *Han’s* criteria, 32 and 13 patients were categorized as ABC type (61.5%) and GCB type (25.0%), respectively. The baseline characteristics at the time of diagnosis are summarized in Table 1.

**Table 1:** DLBCL characteristics at diagnosis (n = 52).

Characteristics	Number of patients (%)
Age, at diagnosis (median)	45 years (range, 17–63)
Sex (male/female)	30 (57.7%)/22 (42.3%)
ECOG-PS score > 1	13 (25.0%)
Ann Arbor stage	
Stage I–II	16 (30.8%)
Stage III–IV	36 (69.2%)
B symptom	15 (28.8%)
> 1 Extranodal site	24 (46.2%)
Bone marrow involvement	13 (25.0%)
Complex karyotypes	8/13 (61.5%)
LDH, at diagnosis (median)	650 IU/L (229–1303)
Elevated LDH	38 (73.1%)
IPI risk	
Low	15 (28.8%)
Low-intermediate	13 (25.0%)
High-intermediate	14 (26.9%)
High	10 (19.2%)
Pathologic diagnosis	
ABC type	32 (61.5%)
GCB type	13 (25.0%)
T-cell/Histiocytic rich	3 (5.8%)
Unclassifiable	3 (5.8%)
Anaplastic variant	1 (1.9%)
Double-Hit	4 (7.7%)

ABC type, activated B-cell type; DLBCL, diffuse large B cell lymphoma; ECOG-PS, Eastern Cooperative Oncology Group-Performance Status; GCB type, germinal center B-cell type; IPI, international prognostic index; LDH, lactate dehydrogenase.

### Allo-HSCT characteristics

Notably, most enrolled patients had previously undergone intensive treatments, and the median number of chemotherapy lines before allo-HSCT was 4 (range, 2–6). Sixteen patients (30.8%) had previously undergone auto-HSCT, and the median time from diagnosis and auto-HSCT to allo-HSCT was 27.1 months (range, 6.2–117.7 months) and 18.5 months (range, 6.5–44.8 months), respectively. The disease status at the time of transplantation was CR (n = 14, 26.9%), PR (n = 16, 30.8%), and active disease (n = 22, 42.3%). Donor types included matched sibling (n = 18, 34.6%), haploidentical (n = 18, 34.6%), matched unrelated (n = 11, 21.2%), and unrelated donors with one allele mismatch (n = 5, 9.6%). The conditioning regimen comprised MAC (n = 2, 3.9%) and RIC (n = 50, 96.1%). Half of the enrolled patients were ABO-type matched (n = 26, 50.0%), 27 (51.9%) were in donor-to-recipient sex mismatch, and 44 (84.6%) were both donor and recipient cytomegalovirus (CMV) IgG seropositive. Table 2 presents the demographic information of the patients who underwent allo-HSCT.

**Table 2:** Allo-HSCT characteristics (n = 52).

Characteristics	Number of patients (%)
Age, recipient at allo-HSCT (median)	45.5 years (range, 17–67)
Age, donor at allo-HSCT (median)	35 years (range, 12–66)
Sex, donor (Male/Female)	39 (75.0%)/13 (25.0%)
Sex mismatched	27 (51.9%)
Female to male	18 (34.6%)
ABO type	
Matched	26 (50.0%)
Major mismatch	10 (19.2%)
Major and minor mismatch	9 (17.3%)
Minor mismatch	7 (13.5%)
ASCT prior to allo-HSCT	16 (30.8%)
Time from ASCT to allo-HSCT (median)	18.5 months (range, 6.5–44.8)
Time from diagnosis to allo-HSCT (median)	27.1 months (range, 6.2–117.7)
Number of lines prior to allo-HSCT (median)	4 lines (range, 2–6)
HCT-CI	
0–1	21 (40.4%)
2	13 (25.0%)
≥ 3	18 (34.6%)
Disease status at allo-HSCT	
Complete response	14 (26.9%)
Partial response	16 (30.8%)
Active disease	22 (42.3%)
CMV IgG seropositivity	
Donor+/Recipient+	44 (84.6%)
Donor−/Recipient+	6 (11.5%)
Donor+/Recipient−	2 (3.8%)
Donor−/Recipient−	0 (0%)
Degree of HLA match	
HLA match (8/8)	29 (55.8%)
HLA mismatch (≤ 7/8 or less)	23 (44.2%)
Donor type	
MSD	18 (34.6%)
Haploidentical	18 (34.6%)
URD full matched	11 (21.2%)
URD one allele mismatched	5 (9.6%)
Conditioning regimen type	
Myeloablative conditioning	2 (3.9%)
Reduced-intensity conditioning	50 (96.1%)
GVHD prophylaxis	
Cyclosporin + methotrexate	18 (34.6%)
Tacrolimus + methotrexate	34 (65.4%)
CD34 × 10^6^/kg infused (median)	7.253 (range, 0.777–16.170)
Median > 20,000/mm^3^ platelet recovery (median)	12 days (range, 6–22)
Median > 500/mm^3^ neutrophil recovery (median)	11 days (range, 9–18)

ASCT, autologous hematopoietic stem-cell transplantation; CMV, cytomegalovirus; GVHD, graft-versus-host disease; HCT-CI, hematopoietic cell transplantation-specific comorbidity index; HLA, human leukocyte antigen; HSCT, hematopoietic stem-cell transplantation; MSD, matched-sibling donor; URD, unrelated donor.

### Response rate, survival outcomes, and complications

**Figure 1:**
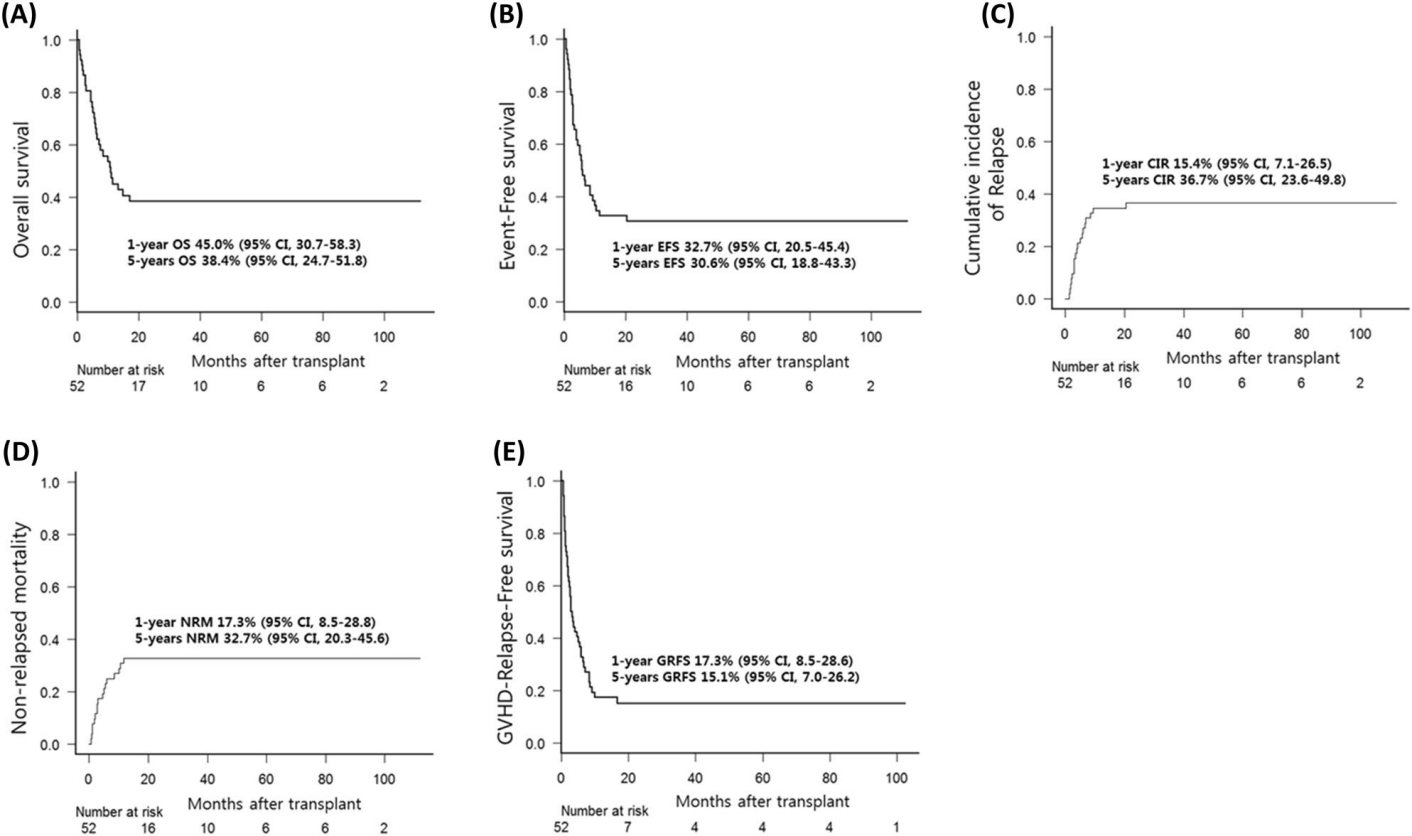
Survival outcomes of patients with relapsed/refractory diffuse large B-cell lymphoma after undergoing allogeneic hematopoietic stem-cell transplantation. The estimated 1- and 5-year (**A**) OS is 45.0% (95% CI, 30.7–58.3) and 38.4% (95% CI, 24.7–51.8). The estimated 1- and 5-year (**B**) EFS is 32.7% (95% CI, 20.5–45.4%) and 30.6% (95% CI, 18.8–43.3). The CIR and NRM at day 100 after allo-HSCT is (**C**) 15.4% (95% CI, 7.1–26.5) and (**D**) 17.3% (95% CI, 8.5–28.8). Furthermore, the estimated 5-year CIR and NRM are 36.7% (95% CI, 23.6–49.8) and 32.7% (95% CI, 20.3–45.6), respectively. The estimated 1- and 5-year (**E**) GRFS is 17.3% (95% CI, 8.5–28.6) and 15.1% (95% CI, 7.0–26.2). CI, confidence interval; CIR, cumulative incidence of relapse; EFS, event-free survival; GRFS, graft-versus-host disease-free, relapse-free survival; HSCT, hematopoietic stem-cell transplantation; NRM, non-relapsed mortality; OS, overall survival; R/R DLBCL, relapsed/refractory diffuse large B-cell lymphoma.

**Figure 2:**
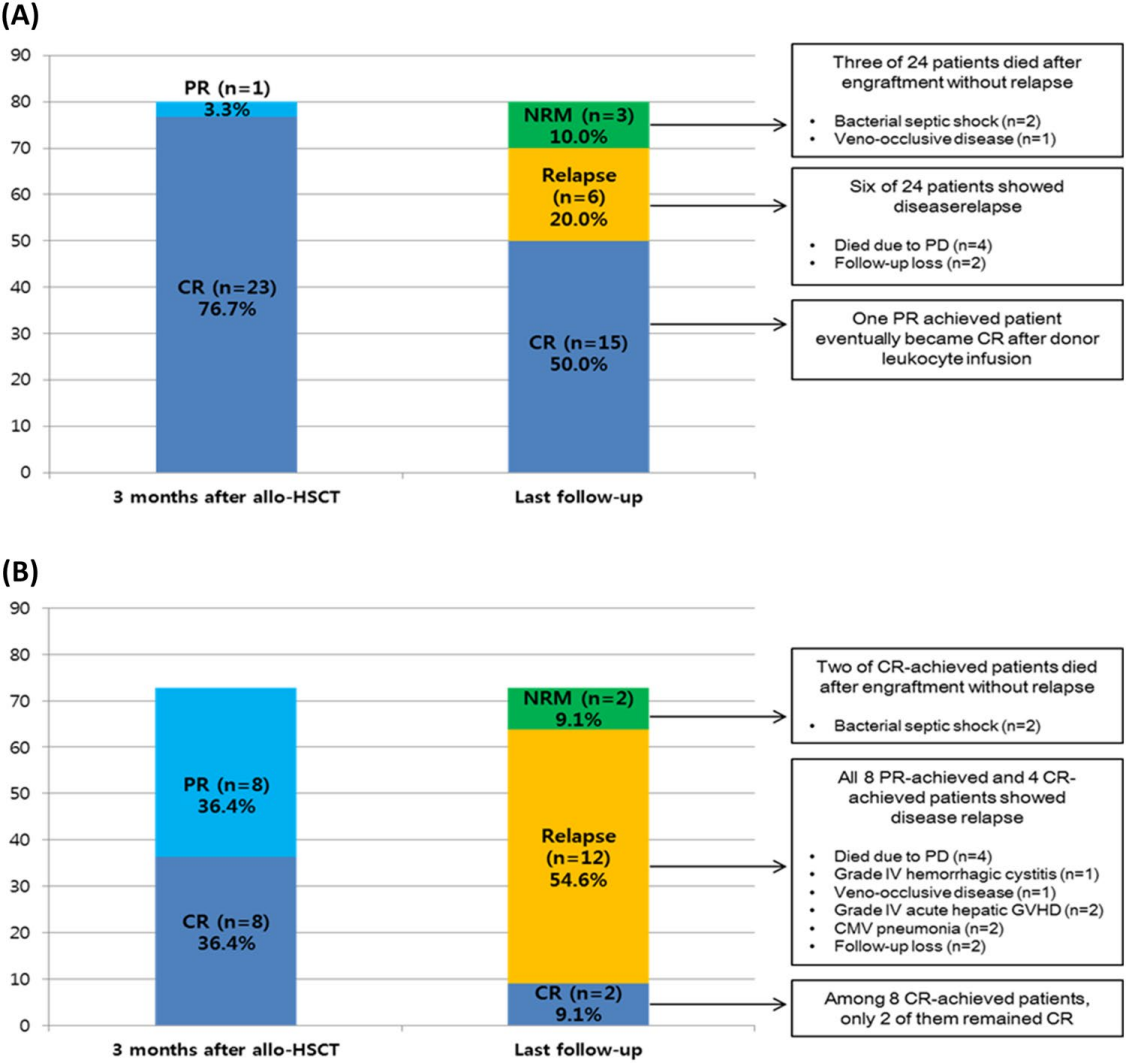
Response rate and clinical course of patients with diffuse relapsed/refractory large B-cell lymphoma after undergoing allogenic hematopoietic stem-cell transplantation. (**A**) Remission-achieved group: Among 30 patients in the remission-achieved group, the overall response rate (23 CR and 1 PR) was 80.0% (n = 24) at 3 months after allo-HSCT. At the last follow-up, 15 patients remained CR (including 1 PR patient who achieved CR after donor leukocyte infusion), but three died after engraftment without relapse, and six experienced DLBCL relapse. (**B**) Active-disease group: Among 22 active-disease group patients, the overall response rate (8 CR and 8 PR) was 72.7% (n = 16) at 3 months after allo-HSCT. However, at the last follow-up, only two patients remained CR, two died after engraftment without relapse, and 12 (4 CR and 6 PR) experienced DLBCL relapse. CMV, cytomegalovirus; CR, complete remission; GVHD, graft-versus-host disease; HSCT, hematopoietic stem-cell transplantation; NRM, non-relapsed mortality; PD, progression of disease; PR, partial remission.

**Figure 3:**
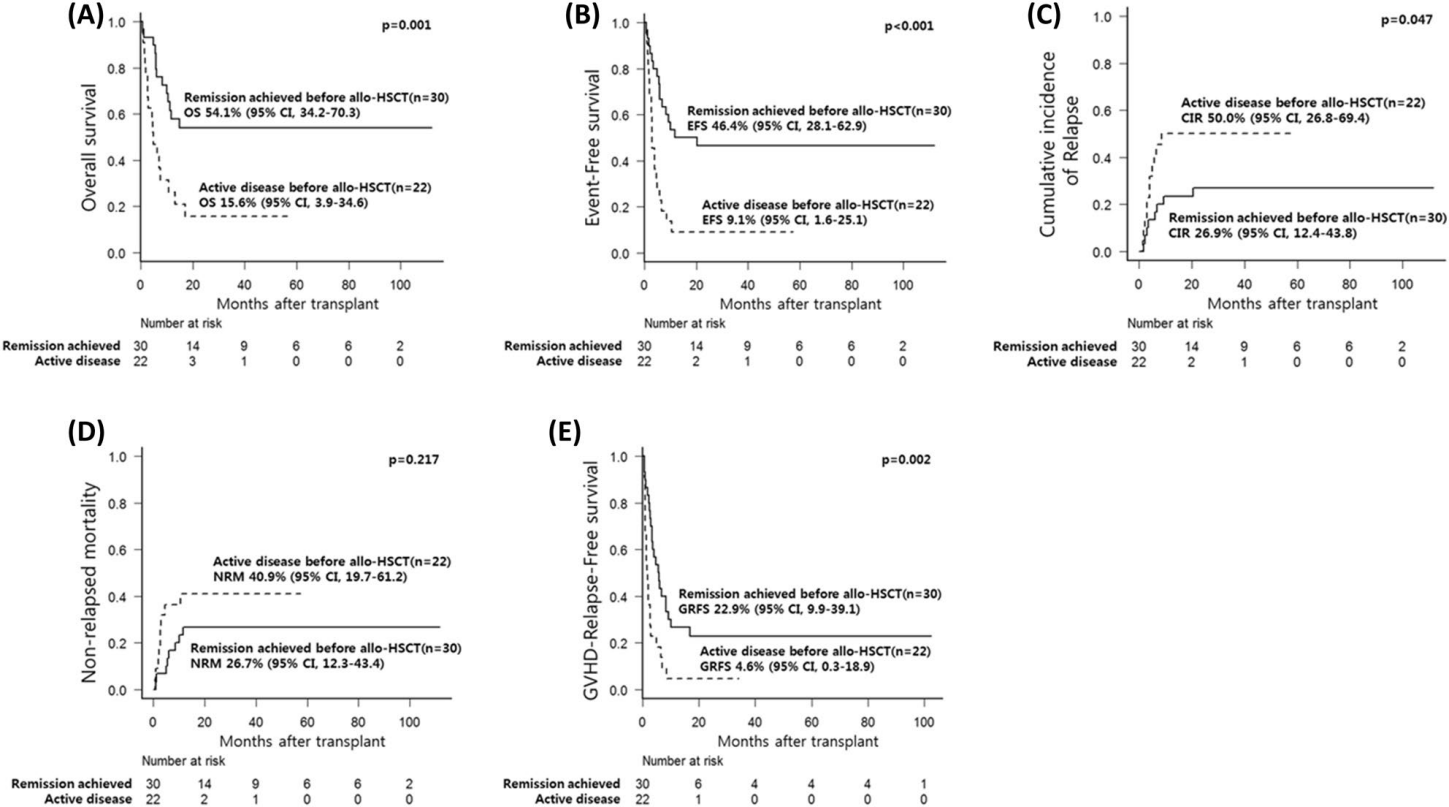
Comparison of survival outcomes between the remission-achieved and active-disease groups in patients with diffuse large B-cell lymphoma who underwent allogeneic hematopoietic stem-cell transplantation. The clinical outcomes of (**A**) OS (54.1% vs. 15.6%, *p* = 0.001), (**B**) EFS (46.4% vs. 9.1%, *p* < 0.001), and (E) GRFS (22.9% vs. 4.6%, *p* = 0.002) were significantly superior in the remission-achieved group with (**C**) lower CIR (26.9% vs. 50.0%, *p* = 0.047), except for (**D**) NRM (26.7% vs. 40.9%, *p* = 0.217). CI, confidence interval; CIR, cumulative incidence of relapse; EFS, event-free survival; GRFS, graft-versus-host disease-free, relapse-free survival; HSCT, hematopoietic stem-cell transplantation; NRM, non-relapsed mortality; OS, overall survival; R/R DLBCL, relapsed/refractory diffuse large B-cell lymphoma.

Over a median follow-up period of 38.3 months (range, 1.9–112.0), the estimated 5-year OS and EFS were 38.4% (95% CI, 24.7–51.8) and 30.6% (95% CI, 18.8–43.3), respectively. The estimated 5-year CIR, NRM, and GRFS were 36.7% (95% CI, 23.6–49.8), 32.7% (95% CI, 20.3–45.6), and 15.1% (95% CI, 6.9–26.2), respectively. Figure 1 shows the 1-year (100 days outcomes in CIR and NRM) and 5-year survival outcomes. Compared to the active-disease group, the remission-achieved group showed a significantly superior rate of CR in the first 3 months after allo-HSCT (76.7% vs. 36.4%, *p* = 0.003) and at the last follow-up (50.0% vs. 13.6%, *p* = 0.006) (Fig. 2). Moreover, except for NRM (26.7% vs. 40.9%, *p* = 0.217), the clinical outcomes of OS (54.1% vs. 15.6%, *p* = 0.001), EFS (46.4% vs. 9.1%, p < 0.001), and GRFS (22.9% vs. 4.6%, *p* = 0.002) were significantly superior in the remission-achieved group with lower CIR (26.9% vs. 50.0%, *p* = 0.047). In detail (Fig. 3), among patients in the remission-achieved group before allo-HSCT (n = 30), 23 achieved CR, one achieved PR, four experienced disease relapse after allo-HSCT and died due to disease progression, and three died after engraftment (two from bacterial septic shock and one from veno-occlusive disease [VOD]) without relapse. In contrast, among patients in the active-disease group before allo-HSCT (n = 22), eight achieved CR. However, only two remained in CR, four experienced disease relapse, and two died due to septic shock during disease remission. All eight of the remaining patients who achieved PR eventually relapsed, and six died after engraftment either due to allo-HSCT-related complications (one with grade IV hemorrhagic cystitis combined with renal failure, one with VOD, and two with grade IV acute hepatic GVHD complicated with liver failure) or infection (two with CMV pneumonia).

The overall cumulative incidence of grade 3 to 4 acute GVHD and moderate-to-severe chronic GVHD that required steroid pulse therapy was 17.3% (95% CI, 8.5–28.8%) and 29.1% (95% CI, 17.2–42.0%), respectively. The active-disease group had a significantly higher incidence of grade 3–4 acute GVHD than the remission-achieved group before allo-HSCT (6.7% vs. 31.8%, *p* = 0.023). However, no significant differences in moderate-to-severe chronic GVHD incidence (37.1% vs. 18.2%, *p* = 0.172) were observed between the two groups. The allo-HSCT-related complication incidence, response rate, and survival outcomes in the remission-achieved and active-disease groups before allo-HSCT are presented in Table 3.

**Table 3:** Allo-HSCT complications and clinical outcomes (n = 52).

Characteristics	Remission achieved before allo-HSCT (n = 30)	Active disease before allo-HSCT (n = 22)	*p*-value
Acute GVHD			
Grade 1–4	30.0% (14.7–46.9)	36.4% (16.9–56.2)	0.530
Grade 3–4	6.7% (1.1–19.5)	31.8% (13.7–51.7)	0.023
Chronic GVHD			
Any grade	54.4% (33.5–71.3)	22.7% (7.0–43.8)	0.267
Moderate to severe	37.1% (19.8–54.5)	18.2% (4.9–38.2)	0.172
Other infectious complications			
Pneumonia	10 (33.3%)	6 (27.3%)	0.640
PJP	3 (10.0%)	3 (13.6%)	0.689
Atypical (pathogen not found)	3 (10.0%)	1 (4.5%)	0.629
Fungal	2 (6.7%)	1 (4.5%)	> 0.999
Viral	2 (6.7%)	1 (4.5%)	> 0.999
BK/JC viral hemorrhagic cystitis	3 (10.0%)	3 (13.6%)	0.689
Bacterial sepsis	4 (13.3%)	2 (9.1%)	> 0.999
Herpes zoster	2 (6.7%)	3 (13.6%)	0.639
Candidiasis	0	1 (4.5%)	0.423
VOD/SOS	1 (3.3%)	1 (4.5%)	> 0.999
Complete response 3 months after allo-HSCT	23 (76.7%)	8 (36.4%)	0.003
Complete response at last follow-up	15 (50.0%)	3 (13.6%)	0.006
Survival outcomes			
Overall survival	54.1% (34.2–70.3)	15.6% (3.9–34.6)	0.001
Event-free survival	46.4% (28.1–62.9)	9.1% (1.6–25.1)	< 0.001
GVHD-free-Relapse-free survival	22.9% (9.9–39.1)	4.6% (0.3–18.9)	0.002
Cumulative incidence of relapse	26.9% (12.4–43.8)	50.0% (26.8–69.4)	0.047
Non-relapsed mortality	26.7% (12.3–43.4)	40.9% (19.7–61.2)	0.217

Allo-HSCT, allogeneic hematopoietic stem-cell transplantation; GVHD, graft-versus-host disease; PJP, *Pneumocystis jirovecii* pneumonia; VOD/SOS, veno-occlusive disease/sinusoidal obstruction syndrome.

### Prognostic factor analysis

**Figure 4:**
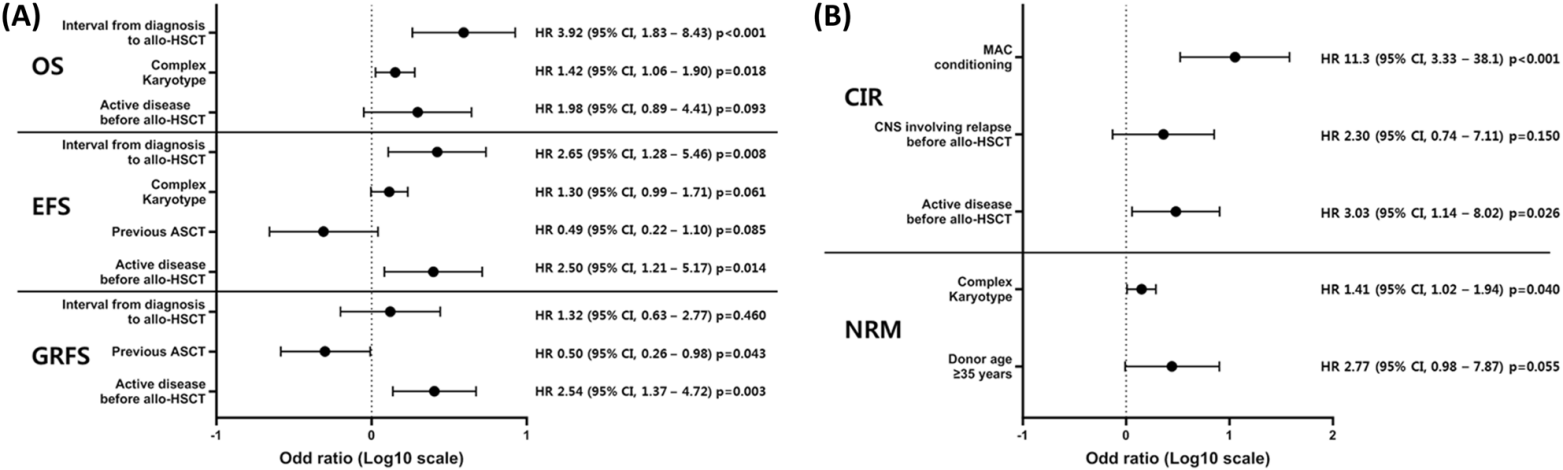
Multivariable analysis of survival outcomes related to allogeneic hematopoietic stem-cell transplantation in patients with relapsed/refractory diffuse large B-cell lymphoma. (**A**) The multivariable analysis for each survival outcome shows that a shorter interval of the median of < 27.1 months from diagnosis to allo-HSCT (HR, 3.92; 95% CI, 1.83–8.43; *p* < 0.001) and complex karyotype presenting bone marrow (HR, 1.42; 95% CI, 1.06–1.90; *p* = 0.018) are significantly related to poor OS. In the case of EFS, a shorter interval compared to the median of < 27.1 months from diagnosis to allo-HSCT (HR, 2.65; 95% CI, 1.28–5.46; *p* = 0.008) and active disease before allo-HSCT (HR, 2.50; 95% CI, 1.21–5.17; *p* = 0.014) are significantly related to poor EFS. Previous ASCT are significantly related to better GRFS (HR, 0.50; 95% CI, 0.26–0.98; *p* = 0.043), but active disease before allo-HSCT are significantly related to poor GRFS (HR, 2.54; 95% CI, 1.37–4.72; *p* = 0.003). (**B**) Active disease before allo-HSCT (HR, 3.03; 95% CI, 1.14–8.02; *p* = 0.026) is also significantly related to higher CIR with MAC conditioning (HR, 11.3; 95% CI, 3.33–38.1; *p* < 0.01). Complex karyotype presenting bone marrow is also related to higher NRM (HR, 1.41; 95% CI, 1.02–1.94; *p* = 0.040). CI, confidence interval; ASCT, autologous stem-cell transplantation; CIR, cumulative incidence of relapse; EFS, event-free survival; GRFS, graft-versus-host disease free, relapse-free survival; HR, hazard ratio; HSCT, hematopoietic stem-cell transplantation; MAC, myeloablative conditioning; NRM, non-relapsed mortality; OS, overall survival; R/R DLBCL, relapsed/refractory diffuse large B-cell lymphoma.

The results of the univariate analysis for OS, EFS, CIR, NRM, and GFRS are presented in Supplementary Tables 1 and 2. In the multivariate analysis, a shorter interval from diagnosis to allo-HSCT (median of < 27.1 months), which reflects relatively rapid disease progression, showed significantly poor OS (hazard ratio [HR], 3.92; 95% CI, 1.83–8.43; *p* < 0.001) and EFS (HR, 2.65; 95% CI, 1.28–5.46; *p* = 0.008). Complex karyotypes in BM involving DLBCL were also associated with poor OS (HR, 1.42; 95% CI, 1.06–1.90; *p* = 0.018) and NRM (HR, 1.41; 95% CI, 1.02–1.94; *p* = 0.040). Active disease before allo-HSCT was associated with significantly lower EFS (HR. 2.50; 95% CI, 1.21–5.17; *p* = 0.014), GFRS (HR, 2.54; 95% CI, 1.37–4.72; *p* = 0.003), and higher CIR (HR, 3.03; 95% CI, 1.14–8.02; *p* = 0.026). The MAC regimen for allo-HSCT was associated with significantly higher CIR (HR, 11.3; 95% CI, 3.33–38.1; *p* < 0.001), and patients with previous autologous stem-cell transplantation (ASCT) had significantly better GRFS (HR, 0.50; 95% CI, 0.26–0.98; *p* = 0.043). The results of the multivariate analysis are presented in Fig. 4.

## Discussion

We demonstrated that allo-HSCT is still a viable treatment option with curative potential for patients with R/R DLBCL with a 3-year OS and EFS of 38.4% and 30.6%, respectively. Compared to the remission-achieved group, patients with active disease before allo-HSCT showed inferior OS, EFS, and GRFS and higher CIR. We preferred to use the RIC regimen for allo-HSCT in R/R DLBCL (n = 50), and the MAC regimen was seldom used (n = 2). Patients who received the MAC regimen showed a higher relapse rate without other survival differences. Further larger studies are required to validate these results. HLA matching degree and GVHD prophylaxis modalities associated with donor type did not influence survival outcomes. A shorter interval (< 27.1 months) between diagnoses to an allo-HSCT, an estimated threshold calculated from the area under the receiver operating characteristic curve months, was associated with inferior OS, thus reflecting rapid progression and/or refractoriness of disease. We also found that complex karyotypes in BM involving DLBCL were associated with worse OS and NRM in the multivariate analysis, which could also be considered evidence of R/R DLBCL aggressiveness, as previously reported^16^. A previously published study by Bento et al.^7^ also showed similar allo-HSCT outcomes in R/R DLBCL with a sufficient follow-up period.

CAR T-cell therapy was initially approved for R/R DLBCL after at least two lines of treatment in chemo-resistant patients and showed promising curative potential. Compared with allo-HSCT, CAR T-cell therapy possesses advantages of safety and lower morbidity and mortality associated with treatment and is indicated for patients with poor performance status^10–13^. All CAR T-cell therapies are performed in a similar manner with an objective response rate of 53–83% and CR rates of 39–58%^10–13^. Furthermore, durable responses to CAR T-cell therapies were demonstrated in a recent update from the ZUMA-1 trial, which showed that 42.6% of patients maintained a response to axicabtagene ciloleucel for over 5 years^22^. In addition, CAR T-cell therapy was introduced more recently in the second-line setting, either for primary refractory or early relapse within 12 months after first-line therapy, and related trials, ZUMA-7, TRANSFORM, and BELINDA, have been reported^23–25^. Both ZUMA-7 and TRANSFORM study results were positive in favor of CAR T-cell therapy compared with conventional treatment using platinum-based salvage chemotherapy, followed by auto-HSCT^23,24^. However, the BELINDA trial resulted in a negative study because of differences in the definition of an event for EFS and lymphodepletion regimens between studies^23–25^. The BELINDA trial also had more patients with pre-infusion PD at 6 weeks than the standard of care group (25.9% vs. 13.8%) and a longer wait time, more than double, than the ZUMA-7 study before CAR T-cell infusion due to allowance of multiple cycles of bridging therapy, requiring recovery periods^23,25^.

As we discussed, CAR T-cell therapy is an attractive treatment option for R/R DLBCL and is associated with favorable clinical outcomes in the elderly and unhealthy populations. However, given the poor prognosis of R/R DLBCL, it is crucial to have access to various alternative treatment options, including allo-HSCT, a new targeted drug such as polatuzumab (anti-CD79), bispecific antibodies, or other immunotherapies. Moreover, with all the treatment options, it is essential to have proper treatment selection individualized to the patients’ characteristics. Among the various treatment modalities, allo-HSCT can be a valuable therapeutic option comparable to CAR T-cell therapy because patients who respond to salvage chemotherapy and achieve CR are less likely to experience distant relapse after successful allo-HSCT engraftment. In our study, the EFS of patients with pre-transplantation CR/PR was 46.4%, and 20 months passed before reaching a plateau after allo-HSCT. The relapse rate was also relatively low in this group at 26.9%. In addition, compared with CAR T-cell therapy, recovery of hematopoietic function is more likely after allo-HSCT and may be an option for patients with complex chromosomal abnormalities in the BM, who generally have poor clinical outcomes. We think that it would be useful to conduct large multicenter studies to investigate this question. Although new therapeutic approaches have been continuously developed and adopted for R/R DLBCL, allo-HSCT remains an attractive therapeutic strategy in multi-drug resistant or R/R DLBCL cases, and manageable chronic GVHD might improve survival outcomes due to the GVL effect without decreasing the quality of life. Allo-HSCT treatment is also available for patients who are currently excluded from CAR T-cell therapy, such as HBsAg carriers, which are commonly available in developing countries.

In cases of relapse or in patients refractory to CAR T-cell therapy, it is difficult to perform follow-up treatment, such as bispecific antibodies, immunochemotherapy, or targeted therapies, owing to the rapid progression of R/R DLBCL. In fact, those salvage treatment responses showed dismal outcomes with only a 3.8% to 14.3% CR rate^26^. In addition, treatment costs will be high if transplantation-related adverse events occur after allo-HSCT. However, in the case of CAR T-cell therapy, the initial cost of infusing manufactured cells is expensive, which might be an insurmountable hurdle for many patients who live in countries unable to reimburse its cost. Moreover, the CAR T-cell manufacturing failure rate may reach 13%, mostly due to suboptimal expansion of CAR T-cells during the manufacturing process^27,28^. If CAR-T cell manufacture failure occurs during lymphodepletion in patients with rapid disease progression, the outcomes will be catastrophic. For patients with DLBCL who are primary refractory or have a large tumor burden, both allo-HSCT and CAR T-cell therapy showed poor treatment outcomes^29,30^. Patients with chemo-susceptible disease after relapse, and they were physically fit transplant candidates, we could employ subsequent CAR T-cell therapy after allo-HSCT treatment failure or relapse. We encountered two patients who relapsed after allo-HSCT and suffered from moderate chronic GVHD, but they successfully underwent CAR T-cell therapy and achieved CR. However, patients with a chemo-resistant disease status or those unfit for transplant have very limited treatment options after CAR T-cell therapy failure with dismal prognosis. Overall, for patients with a high tumor burden and rapid progression after salvage chemotherapy, both treatment modalities have definite limitations, and patients predicted to show poor prognosis after allo-HSCT may also expect to show poor prognosis after CAR T-cell therapy.

This study had several limitations. First, it was retrospective in nature, with the treating physician deciding the treatment option with limited available salvage regimens. Second, the relatively small sample size led to less reliable statistical analysis results, and it was challenging to deduce any general conclusions. Third, the introduction of CAR T-cell therapy in Korea was later than in other countries because of regulatory issues associated with cellular therapies. Among the CAR T-cell therapies, only tisagenlecleucel was officially approved by the Korean Ministry of Food and Drug Safety in 2021 and covered by National Health Insurance in April 2022. Before then, CAR T-cell therapy could only be applied to selected patients with R/R DLBCL through clinical trial enrollment. Therefore, there were few treatment options for patients with R/R DLBCL, and allo-HSCT was a reliable choice as an established treatment for R/R NHL. Moreover, even after tisagenlecleucel became available as a third-line treatment, only a limited number of institutions were able to administer the treatment. Furthermore, many developing countries may also be unable to utilize novel treatment, including CAR T-cell therapy, in frontline or salvage settings.

In summary, allo-HSCT still has a role in the CAR T-cell therapy era, with acceptable OS and EFS rates in patients who achieve CR/PR after salvage treatment. Therefore, allo-HSCT could be considered a treatment modality with curable potential, especially in young patients with chemo-susceptible disease who are fit for intensive therapy. Allo-HSCT is an established treatment modality, and some patients achieve a cure. Patients undergoing CAR T-cell therapy have improved treatment outcomes; however, the current follow-up period is relatively short, and longer-term studies are needed. Further well-designed prospective studies are needed to confirm the prognostic factors and investigate the optimal treatment for R/R DLBCL and to compare the clinical outcomes of allo-HSCT and CAR T-cell therapy head-to-head.

### Supplementary Information

Supplementary Tables.

## Data Availability

The data that support the findings of this study are available upon request from the corresponding author. The data are not publicly available because of privacy or ethical restrictions.
