# Supplementary material for: The salvage role of allogeneic hematopoietic stem-cell transplantation in relapsed/refractory diffuse large B cell lymphoma
Source: Sci Rep. 2023 Oct 15;13:17496. doi: 10.1038/s41598-023-44241-0 (PMC10577125; doi:10.1038/s41598-023-44241-0)
Supplement: Supplementary file 1 — Supplementary Tables. [file 41598_2023_44241_MOESM1_ESM.docx]

# The salvage role of allogeneic hematopoietic stem- cell transplantation in relapsed/refractory diffuse large B cell lymphoma

# Supplementary Table 1. Univariate analysis of survival outcomes in 52 patients with allo-HSCT

| **Variables** † | **OS (95% CI)** | ***p-value*** | **EFS (95% CI)** | ***p*-value** | **CIR* (95% CI)** | ***p*-value** | **NRM (95% CI)** | ***p*-value** |
| --- | --- | --- | --- | --- | --- | --- | --- | --- |
| **Recipient age at allo-HSCT (years)** |  | 0.901 |  | 0.684 |  | 0.134 |  | 0.193 |
| < 45 years (n=26) | 36.8% (18.1–55.8) |  | 30.8% (14.6–48.5) |  | 46.2% (26.0–64.2) |  | 23.1% (9.0–40.9) |  |
| ≥ 45 years (n=26) | 40.1% (21.4–58.3) |  | 30.3% (14.1–48.2) |  | 27.4% (11.7–45.8) |  | 42.3% (22.9–60.5) |  |
| **Recipient sex** |  | 0.352 |  | 0.197 |  | 0.473 |  | 0.540 |
| Male (n=30) | 31.2% (15.1–48.8) |  | 23.3% (10.3–39.4) |  | 32.3% (13.7–52.6) |  | 36.7% (19.6–54.0) |  |
| Female (n=22) | 47.4% (25.3–66.6) |  | 40.4% (20.3–59.8) |  | 40.0% (22.3–57.1) |  | 27.3% (10.7–47.0) |  |
| **Interval from diagnosis to allo-HSCT** |  | <0.001 |  | <0.001 |  | 0.124 |  | 0.120 |
| ≥ 26 months (n=28) | 58.4% (37.5–74.5) |  | 46.4% (27.6–63.3) |  | 28.6% (13.2–46.0) |  | 25.0% (10.8–42.2) |  |
| < 26 months (n=24) | 11.9% (2.2–30.4) |  | 12.5% (3.1–28.7) |  | 45.8% (24.6–64.8) |  | 41.7% (21.3–60.9) |  |
| **Pathologic diagnosis** |  | 0.963 |  | 0.763 |  | 0.236 |  | 0.607 |
| ABC type (n=32) | 35.5% (18.9–52.6) |  | 25.0% (11.8–40.7) |  | 43.8% (26.0–60.2) |  | 31.2% (16.0–47.8) |  |
| Others (n=20) | 42.7% (20.5–63.2) |  | 40.0% (19.3–60.0) |  | 25.0% (8.6–45.7) |  | 35.0% (15.0–55.9) |  |
| **Initial IPI** |  | 0.187 |  | 0.096 |  | 0.388 |  | 0.499 |
| 0–2 (n=28) | 46.8% (27.1–64.2) |  | 39.3% (21.7–56.5) |  | 32.1% (15.7–49.8) |  | 28.6% (13.2–46.1) |  |
| 3–5 (n=24) | 27.6% (10.8–47.4) |  | 20.8% (7.6–38.5) |  | 41.7% (21.5–60.7) |  | 37.5% (18.2–56.8) |  |
| **BM involvement** |  | 0.263 |  | 0.390 |  | 0.297 |  | 0.063 |
| No (n=39) | 40.8% (24.5–56.4) |  | 33.1% (19.1–47.9) |  | 41.2% (25.5–56.3) |  | 25.6% (13.1–40.2) |  |
| Yes (n=13) | 30.8% (9.5–55.4) |  | 23.1% (5.6–47.5) |  | 23.1% (4.8–49.2) |  | 53.8% (22.2–77.5) |  |
| **Chromosomal abnormalities** |  | 0.030 |  | 0.024 |  | 0.796 |  | 0.047 |
| Normal karyotype (n=44) | 43.4% (27.8–58.0) |  | 36.2% (22.4–50.2) |  | 36.5% (22.4–50.7) |  | 27.3% (15.0–41.0) |  |
| Complex karyotype (n=8) | 12.5% (0.7–42.3) |  | 0% |  | 37.5% (6.1–70.9) |  | 62.5% (10.8–90.5) |  |
| **HLA matching degree** |  | 0.345 |  | 0.414 |  | 0.874 |  | 0.360 |
| HLA match (8/8) (n=29) | 41.4% (22.4–59.5) |  | 34.5% (18.2–51.4) |  | 37.9% (20.4–55.4) |  | 27.6% (12.7–44.8) |  |
| HLA mismatch (≤7/8 or less) (n=23) | 34.8% (16.6–53.7) |  | 25.4% (9.9–44.3) |  | 35.5% (16.2–55.5) |  | 39.1% (19.2–58.7) |  |
| **Chemotherapy lines prior to allo-HSCT** |  | 0.511 |  | 0.701 |  | 0.482 |  | 0.204 |
| < 4 (n=22) | 44.7% (22.5–64.8) |  | 36.4% (17.4–55.7) |  | 40.9% (20.1–60.8) |  | 22.7% (7.9–42.1) |  |
| ≥ 4 (n=30) | 34.3% (17.9–51.4) |  | 26.3% (12.2–42.7) |  | 33.8% (17.2–51.1) |  | 40.0% (22.3–57.1) |  |
| **Previous ASCT** |  | 0.061 |  | 0.015 |  | 0.202 |  | 0.327 |
| No (n=36) | 30.7% (15.9–46.8) |  | 21.9% (10.1–36.5) |  | 42.0% (25.3–57.8) |  | 36.1% (20.7–51.8) |  |
| Yes (n=16) | 54.2% (27.1–75.0) |  | 50.0% (24.5–71.0) |  | 25.0% (7.2–48.1) |  | 25.0% (7.2–48.2) |  |
| **Disease status before allo-HSCT** |  | 0.001 |  | <0.001 |  | 0.047 |  | 0.217 |
| CR/PR (n=30) | 54.1% (34.2–70.3) |  | 46.4% (28.1–62.9) |  | 26.9% (12.4–43.8) |  | 26.7% (12.3–43.4) |  |
| SD/PD (n=22) | 15.6% (3.9–34.6) |  | 9.1% (1.6–25.1) |  | 50.0% (26.8–69.4) |  | 40.9% (19.7–61.2) |  |
| **Conditioning intensity** |  | 0.526 |  | 0.212 |  | 0.014 |  | 0.453 |
| RIC (n=50) | 40.2% (26.1–53.9) |  | 31.9% (19.6–44.9) |  | 34.1% (21.2–47.4) |  | 34.0% (21.2–47.3) |  |
| MAC (n=2) | 0% |  | 0% |  | 100% |  | 0% |  |
| **GVHD prophylaxis** |  | 0.377 |  | 0.305 |  | 0.681 |  | 0.558 |
| Cyclosporin + methotrexate (n=18) | 44.4% (20.3–66.2) |  | 38.9% (17.5–60.0) |  | 33.3% (12.9–55.4) |  | 27.8% (9.5–49.8) |  |
| Tacrolimus + Methotrexate (n=34) | 35.4% (19.5–51.7) |  | 26.1% (12.9–41.6) |  | 38.6% (22.1–54.8) |  | 35.3% (19.6–51.4) |  |
| **ABO matching degree** |  | 0.762 |  | 0.966 |  | 0.333 |  | 0.527 |
| Match and minor mismatch (n=33) | 37.3% (20.4–54.2) |  | 26.9% (13.3–42.7) |  | 42.8% (25.3–59.2) |  | 30.3% (15.5–46.5) |  |
| Major and major/minor mismatch (n=19) | 39.5% (17.9–60.5) |  | 36.8% (16.5–57.5) |  | 26.3% (9.0–47.6) |  | 36.8% (15.7–58.3) |  |
| **CNS involving relapse before allo-HSCT** |  | 0.502 |  | 0.383 |  | 0.045 |  | 0.282 |
| No (n=45) | 41.0% (26.1–55.2) |  | 31.7% (18.6–45.5) |  | 31.3% (18.2–45.2) |  | 35.6% (21.8–49.6) |  |
| Yes (n=7) | 19.0% (0.9–55.6) |  | 25.0% (3.7–55.8) |  | 71.4% (15.1–94.2) |  | 14.3% (0.4–50.3) |  |
| **HCT–CI** |  | 0.451 |  | 0.886 |  | 0.420 |  | 0.529 |
| 0–2 (n=34) | 41.6% (24.1–58.2) |  | 29.4% (15.4–44.9) |  | 41.2% (24.3–57.3) |  | 29.4% (15.1–45.3) |  |
| ≥ 3 (n=18) | 32.4% (12.7–54.0) |  | 33.3% (13.7–54.5) |  | 27.8% (9.5–49.8) |  | 38.9% (16.5–60.9) |  |
| **Donor to recipient sex mismatch** |  | 0.197 |  | 0.249 |  | 0.059 |  | 0.429 |
| Matched (n=25) | 26.5% (10.8–45.3) |  | 24.0% (9.8–41.7) |  | 48.0% (27.1–66.2) |  | 28.0% (11.9–46.8) |  |
| Mismatched (n=27) | 49.0% (28.6–66.6) |  | 36.7% (19.2–54.3) |  | 26.3% (11.2–44.2) |  | 37.0% (19.2–55.0) |  |
| **CMV profile** |  | 0.358 |  | 0.383 |  | 0.957 |  | 0.623 |
| R+/D+ (n=44) | 41.0% (25.8–55.7) |  | 31.7% (18.6–45.5) |  | 36.5% (22.4–50.8) |  | 31.8% (18.6–45.9) |  |
| R+/D− or R-−/D+ (n=8) | 25.0% (3.7–55.8) |  | 25.0% (3.7–55.8) |  | 37.5% (6.1–71.0) |  | 37.5% (7.2–69.4) |  |
| **Donor age** |  | 0.270 |  | 0.915 |  | 0.101 |  | 0.050 |
| < 35 years (n=26) | 50.0% (28.9–67.9) |  | 34.2% (17.0–52.2) |  | 46.6% (26.2–64.7) |  | 19.2% (6.7–36.5) |  |
| ≥ 35 years (n=26) | 28.3% (12.6–46.4) |  | 26.9% (11.9–44.5) |  | 26.9% (11.5–45.1) |  | 46.2% (25.9–64.2) |  |
| **CD34 dosage** |  | 0.470 |  | 0.839 |  | 0.402 |  | 0.657 |
| < 7.25×10^6^/kg (n=26) | 30.2% (13.6–48.7) |  | 26.9% (11.9–44.5) |  | 42.3% (22.9–60.5) |  | 30.8% (14.1–49.3) |  |
| ≥ 7.25×10^6^/kg (n=26) | 46.3% (25.7–64.6) |  | 34.2% (17.0–52.2) |  | 31.2% (14.3–49.8) |  | 34.6% (17.0–53.0) |  |
| **Acute GVHD Gr. III–IV on day 100** |  | 0.262 |  | 0.364 |  | 0.578 |  | 0.912 |
| 0–2 (n=43) | 40.6% (25.4–55.3) |  | 32.4% (19.1–46.4) |  | 35.0% (21.0–49.4) |  | 32.6% (19.0–46.8) |  |
| 3–4 (n=9) | 25.9% (3.9–57.0) |  | 22.2% (3.4–51.3) |  | 44.4% (10.5–74.7) |  | 33.3% (6.1–64.9) |  |
| **Chronic GVHD moderate to severe** |  | 0.070 |  | 0.069 |  | 0.570 |  | 0.157 |
| None to mild (n=37) | 31.9% (16.9–48.1) |  | 24.3% (12.1–38.8) |  | 37.8% (22.2–53.4) |  | 37.8% (22.2–53.3) |  |
| Moderate to severe (n=15) | 53.3% (26.3–74.4) |  | 46.7% (21.2–68.7) |  | 33.3% (11.3–57.4) |  | 20.0% (4.4–43.6) |  |

ASCT, autologous hematopoietic stem cell transplantation; BM, bone marrow; CIR, cumulative incidence of relapse; CMV, cytomegalovirus; CNS, central nervous system; EFS, event-free survival; GVHD, graft versus host disease; HCT-CI, hematopoietic cell transplantation-specific comorbidity index; HLA, human leukocyte antigen; HSCT, hematopoietic stem cell transplantation; IPI, international prognostic index; MSD, matched sibling donor; NRM, non-relapsed mortality; OS, overall survival; URD, unrelated donor

† Univariate analysis variables were selected based on previous literature on currently known or potential factors affecting survival outcomes according to the researcher's prediction.

* Any effusion represents either pleural effusion or ascites.

# Supplementary Table 2. Univariate analysis of GRFS in 52 patients with allo-HSCT

| **Variables** † | **GRFS (95% CI)** | ***p-value*** | **GRFS (95% CI)** | ***p*-value** |
| --- | --- | --- | --- | --- |
| **Recipient age at allo-HSCT (years)** |  | 0.191 |  |  |
| < 45 years (n=26) | 11.5% (2.9–26.7) |  |  |  |
| ≥ 45 years (n=26) | 19.2% (7.0–36.0) |  |  |  |
| **Recipient sex** |  | 0.577 |  |  |
| Male (n=30) | 10.0% (2.6–23.6) |  |  |  |
| Female (n=22) | 22.7% (8.3–41.4) |  |  |  |
| **Interval from diagnosis to allo-HSCT** |  | 0.006 |  | 0.460 |
| ≥ 26 months (n=28) | 21.4% (8.7–37.8) |  | 1.00 |  |
| < 26 months (n=24) | 8.3% (1.4–23.3) |  | 1.32 (0.63–2.77) |  |
| **Pathologic diagnosis** |  | 0.662 |  |  |
| ABC type (n=32) | 15.6% (5.7–30.0) |  |  |  |
| Others (n=20) | 13.3% (2.7–32.7) |  |  |  |
| **Initial IPI** |  | 0.311 |  |  |
| 0–2 (n=28) | 21.4% (8.7–37.8) |  |  |  |
| 3–5 (n=24) | 8.3% (1.4–23.3) |  |  |  |
| **BM involvement** |  | 0.454 |  |  |
| No (n=39) | 17.6% (7.6–31.0) |  |  |  |
| Yes (n=13) | 7.7% (0.5–29.2) |  |  |  |
| **Chromosomal abnormalities** |  | 0.124 |  |  |
| Normal karyotype (n=44) | 17.9% (8.3–30.5) |  |  |  |
| Complex karyotype (n=8) | 0% |  |  |  |
| **HLA matching degree** |  | 0.812 |  |  |
| HLA match (8/8) (n=29) | 13.8% (4.4–28.6) |  |  |  |
| HLA mismatch (≤7/8 or less) (n=23) | 17.4% (5.4–35.0) |  |  |  |
| **Chemotherapy lines prior to allo-HSCT** |  | 0.984 |  |  |
| < 4 (n=22) | 18.2% (5.7–36.3) |  |  |  |
| ≥ 4 (n=30) | 12.5% (3.6–27.3) |  |  |  |
| **Previous ASCT** |  | 0.029 |  | 0.043 |
| No (n=36) | 13.9% (5.1–27.1) |  | 1.00 |  |
| Yes (n=16) | 18.8% (4.6–40.2) |  | 0.50 (0.26–0.98) |  |
| **Disease status before allo-HSCT** |  | 0.002 |  | 0.003 |
| CR/PR (n=30) | 22.9% (9.9–39.1) |  | 1.00 |  |
| SD/PD (n=22) | 4.6% (0.3–18.9) |  | 2.54 (1.37–4.72) |  |
| **Conditioning intensity** |  | 0.260 |  |  |
| RIC (n=50) | 15.7% (7.3–27.2) |  |  |  |
| MAC (n=2) | 0% |  |  |  |
| **GVHD prophylaxis** |  | 0.431 |  |  |
| Cyclosporin + methotrexate (n=18) | 16.7% (4.1–36.5) |  |  |  |
| Tacrolimus + methotrexate (n=34) | 14.1% (4.9–28.1) |  |  |  |
| **ABO matching degree** |  | 0.761 |  |  |
| Match and minor mismatch (n=33) | 15.2% (5.5–29.2) |  |  |  |
| Major and major/minor mismatch (n=19) | 15.8% (3.9–34.9) |  |  |  |
| **CNS involving relapse before allo-HSCT** |  | 0.904 |  |  |
| No (n=45) | 15.2% (6.6–27.3) |  |  |  |
| Yes (n=7) | 14.3% (0.7–46.5) |  |  |  |
| **HCT–CI** |  | 0.908 |  |  |
| 0–2 (n=34) | 14.7% (5.4–28.5) |  |  |  |
| ≥ 3 (n=18) | 16.7% (4.1–36.5) |  |  |  |
| **Donor to recipient sex mismatch** |  | 0.226 |  |  |
| Matched (n=25) | 8.0% (1.4–22.5) |  |  |  |
| Mismatched (n=27) | 22.2% (9.0–39.0) |  |  |  |
| **CMV profile** |  | 0.430 |  |  |
| R+/D+ (n=44) | 15.6% (6.7–27.8) |  |  |  |
| R+/D− or R−/D+ (n=8) | 12.5% (0.7–42.3) |  |  |  |
| **Donor age** |  | 0.627 |  |  |
| < 35 years (n=26) | 14.4% (4.1–30.9) |  |  |  |
| ≥ 35 years (n=26) | 15.4% (4.8–31.5) |  |  |  |
| **CD34 dosage** |  | 0.338 |  |  |
| < 7.25 × 10^6^/kg (n=26) | 15.4% (4.8–31.5) |  |  |  |
| ≥ 7.25 × 10^6^/kg (n=26) | 15.4% (4.8–31.5) |  |  |  |

ASCT, autologous hematopoietic stem cell transplantation; BM, bone marrow; CMV, cytomegalovirus; CNS, central nervous system; GRFS, graft-versus-host disease-free, relapse-free survival; HCT-CI, hematopoietic cell transplantation-specific comorbidity index; HLA, human leukocyte antigen; HSCT, hematopoietic stem cell transplantation; IPI, international prognostic index; MSD, matched sibling donor; URD, unrelated donor

† Univariate analysis variables were selected based on previous literature on currently known or potential factors affecting survival outcomes according to the researcher's prediction.

* Any effusion represents either pleural effusion or ascites.
